# From neuroinflammation to neuroprotection: a bibliometric analysis of anesthesia-associated postoperative cognitive dysfunction to guide clinical research (2000–2024)

**DOI:** 10.3389/fmed.2026.1822901

**Published:** 2026-04-22

**Authors:** Xingcen Wang, Zhiwei Yang, Ting Tang, Huijuan Huang, Jianzhi Shi, Jie He, Hua Zhang

**Affiliations:** Department of Anesthesiology and Pain Management, Hangzhou Third People’s Hospital, Hangzhou, China

**Keywords:** anesthesia, bibliometric analysis, hotspots, neuroinflammation, perioperative neurocognitive disorders, POCD, postoperative cognitive dysfunction, research trends

## Abstract

**Introduction:**

Postoperative cognitive dysfunction is a common complication following anesthesia and surgery, particularly in elderly patients, yet its pathophysiology and optimal prevention strategies remain incompletely understood. This study employs bibliometric methods to analyze research trends in anesthesia-associated POCD from 2000 to 2024.

**Methods:**

A systematic review of 923 publications from the Web of Science Core Collection, cross-validated with the PubMed database, was conducted. Bibliometric analyses were performed using the R package “bibliometrix,” VOSviewer, and CiteSpace to evaluate publication trends, key contributors, collaborative networks, co-citation patterns, and keyword evolution.

**Results:**

Research output has grown steadily since 2000, with notable acceleration after 2018. The United States leads in productivity and influence, followed by China and Germany. Duke University is the most prolific institution. The British Journal of Anaesthesia and Anesthesiology are the core journals in this field. Keyword analysis reveals an evolution from early focus on surgical types and cognitive assessment toward neuroinflammation as the central pathological mechanism, with increasing attention to the delirium-POCD continuum, anesthetic optimization, and multimodal prevention. Emerging frontiers include the intersection of POCD with Alzheimer’s disease pathology, the role of the gut-brain axis, and the translation of mechanistic insights into targeted neuroprotective strategies.

**Conclusion:**

This bibliometric analysis delineates the evolution of POCD research from descriptive epidemiology to mechanistic and translational inquiry. Neuroinflammation has emerged as the unifying pathological hub. Key challenges include heterogeneity in diagnostic criteria, difficulty isolating anesthesia effects from surgical trauma, and the gap between preclinical findings and clinical efficacy. Future research priorities should focus on harmonizing diagnostic standards, validating biomarkers, and conducting large-scale multi-center trials to translate mechanistic discoveries into perioperative brain health strategies.

## 1 Introduction

Postoperative cognitive dysfunction (POCD) describes a recognized decline in cognitive domains such as memory, attention, executive function, and processing speed following surgical procedures performed under anesthesia (1). With the demographic shift towards an aging population and advancements in surgical techniques, an increasing number of elderly patients undergo major operations, making POCD a significant public health concern. Reported incidence rates vary widely but can affect up to one-third of older surgical patients, particularly after major procedures like cardiac or orthopedic surgery (2, 3). The consequences of POCD extend beyond transient confusion; it is associated with prolonged hospital stays, increased healthcare costs, heightened dependency, elevated long-term morbidity and mortality, and a profound reduction in quality of life for patients and their families (4–6).

The etiology of POCD is unequivocally multifactorial and complex. Patient-specific vulnerabilities play a crucial role, with advanced age, lower educational attainment, and pre-existing cognitive impairment being well-established non-modifiable risk factors (7). Genetic susceptibility, such as the presence of the Apolipoprotein E ε4 allele, further compounds individual risk (8). From a perioperative perspective, the type and magnitude of surgical trauma (e.g., use of cardiopulmonary bypass), the choice and depth of anesthesia, and the quality of postoperative pain management are all implicated (9–12).

At its core, the prevailing pathophysiological hypothesis centers on neuroinflammation. The “double-hit” model posits that the systemic inflammatory response triggered by surgical trauma, compounded by potential direct or indirect effects of anesthetic agents, can disrupt the blood–brain barrier, activate resident microglia and astrocytes, and initiate a cascade of central nervous system inflammation (13, 14). This inflammatory milieu is cytotoxic to neurons, impairs synaptic plasticity, and is believed to serve as a critical biological bridge linking the acute perioperative insult to both transient cognitive deficits and the potential acceleration of underlying chronic neurodegenerative processes, such as Alzheimer’s disease (15–17).

Despite over two decades of intensive research, significant challenges remain. The debate continues regarding the relative contributions of anesthesia per se versus surgical stress and other perioperative factors (18, 19). Methodological heterogeneity in defining and assessing POCD has historically hampered cross-study comparisons and consensus building (20), a challenge partially addressed by recent efforts to standardize nomenclature, such as the introduction of the umbrella term “perioperative neurocognitive disorders” (21). Furthermore, while numerous potential neuroprotective strategies have been explored—from pharmacological agents like dexmedetomidine to non-pharmacological approaches like regional anesthesia techniques or cognitive prehabilitation—definitive, widely applicable preventive or therapeutic interventions are still lacking (22–24).

Consequently, the scientific literature on anesthesia-associated POCD has expanded rapidly and diversely across disciplines including anesthesiology, neurology, geriatrics, and immunology. This growth underscores the need for a comprehensive synthesis to map the intellectual landscape, identify knowledge gaps, and guide future research directions. Bibliometric analysis offers a powerful, quantitative approach to achieve this, systematically evaluating publication trends, key contributors, collaborative networks, and the evolution of research themes. Unlike traditional narrative reviews, bibliometric methods enable unbiased, reproducible identification of emerging hotspots and structural patterns within large literature corpora, making them particularly suitable for mature yet rapidly evolving fields like POCD research. This study aims to provide such a holistic overview through a bibliometric analysis of the global research output on POCD from 2000 to 2024, delineating its past trajectory, current hotspots, and potential future frontiers.

## 2 Methods

### 2.1 Data source and search strategy

This bibliometric analysis relied exclusively on the Web of Science Core Collection (WoSCC), a premier database recognized for its rigorous indexing of high-impact scientific literature. To ensure methodological consistency and data quality, the search was confined to three core citation indices: Science Citation Index Expanded (SCI-EXPANDED), Social Sciences Citation Index (SSCI), and Emerging Sources Citation Index (ESCI). Data retrieval was performed on 10 January 2024.

A 25-year timeframe from 1 January 2000 to 31 December 2024 was examined to capture the complete modern research trajectory of anesthesia-associated postoperative cognitive dysfunction (POCD). The search strategy was iteratively refined by integrating domain-specific terminology. The final query, executed in the advanced search module of WoS, targeted the “Topic” field (encompassing titles, abstracts, author keywords, and Keywords Plus).

### 2.2 Inclusion and exclusion criteria

To align the dataset with the study’s objectives, publications were subjected to predefined eligibility criteria. The analysis was limited to original research articles and review articles published in English. Publications falling into the following categories were systematically excluded: non-peer-reviewed document types, including conference abstracts, editorials, letters, books, studies whose primary focus was unrelated to the clinical or mechanistic scope of POCD (such as purely animal-based preclinical research), and duplicate entries.

TS = (“postoperative cognitive dysfunction” OR “postoperative cognitive decline” OR “postoperative cognitive impairment”) AND TS = (anesthesia OR anaesthesia OR surgery) NOT TS = (animal OR rat OR mouse).

### 2.3 Data selection

A structured, multi-phase screening protocol was implemented by two independent investigators to refine the dataset. The process commenced with an automated filtration step using WoS native tools to restrict the initial 1,886 records to the document types of “Article” and “Review” in English, yielding 923 publications.

Subsequently, a subject-category refinement was applied. To concentrate on the most pertinent disciplinary perspectives, these 923 records were filtered to include only those classified under the Web of Science categories of Anesthesiology, Surgery, Neurosciences, and Clinical Neurology. Records assigned to multiple relevant categories were merged, and intra-dataset duplicates were identified and removed, resulting in a consolidated library.

**Figure 1:**
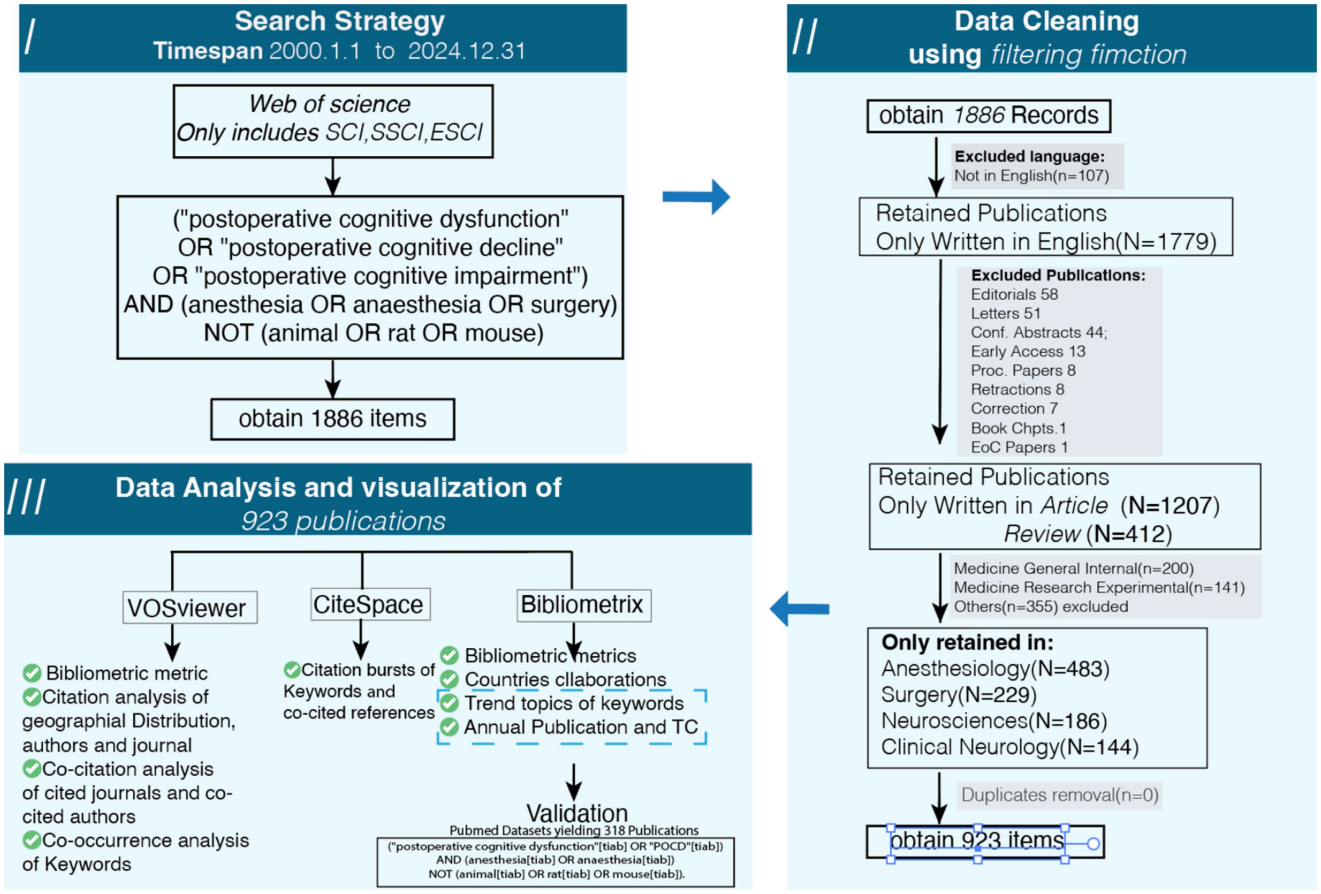
Flowchart of the publications screening and analysis process.

Finally, a manual relevance screening was conducted. The titles and abstracts of all records in the consolidated library were examined to ensure thematic alignment with anesthesia-associated POCD. Any discordance in judgment between the two primary screeners was resolved through consensus discussion or, if needed, arbitration by a third senior researcher. The entire screening and selection workflow is depicted in a PRISMA-compliant flow diagram (Figure 1). Following this rigorous process, a final corpus comprising 923 publications was established as the basis for all subsequent bibliometric analyses.

### 2.4 Cross-database validation strategy

To ensure the robustness and generalizability of our findings beyond the Web of Science Core Collection (WoSCC), and to address potential database-specific biases, we conducted a systematic validation using the PubMed database. PubMed was selected for its complementary role in biomedicine, offering extensive coverage of clinical studies, trial registrations, and literature indexed with detailed Medical Subject Headings (MeSH), which can capture nuanced clinical themes. To maintain methodological comparability with the WoSCC search, we adapted the core search syntax for PubMed’s query system. The final PubMed query was: (“postoperative cognitive dysfunction”[tiab] OR “POCD”[tiab]) AND (anesthesia[tiab] OR anaesthesia[tiab]) NOT (animal[tiab] OR rat[tiab] OR mouse[tiab]).

The search was restricted to the same timeframe (January 1, 2000 – December 31, 2024) and to English-language publications. To enhance clinical relevance and data quality, we applied the following filters: (1) records must have an abstract available; (2) study species was limited to human; (3) preprints were excluded; (4) only the following publication types were included: Comparative Study, Controlled Clinical Trial, Meta-Analysis, Multicenter Study, Observational Study, Randomized Controlled Trial, Review, and Systematic Review.

Data from the resulting PubMed corpus were extracted and analyzed in parallel with the WoSCC data. We performed identical bibliometric analyses, including annual publication trend analysis, high-frequency keyword extraction, and keyword co-occurrence network analysis using VOSviewer (with consistent threshold settings). To quantitatively assess the thematic consistency between the two databases, we calculated the Jaccard similarity coefficient based on the overlap of the top 50 keywords from each corpus. Comparative visualization of keyword networks and temporal trends was conducted to evaluate structural and dynamic alignment.

Applying these criteria, the initial search results were refined to a final validation corpus of 318 publications from PubMed.

### 2.5 Data analysis

The analytical phase employed a suite of specialized software tools to extract, quantify, and visualize patterns within the literature corpus.

Descriptive bibliometric statistics and the evolution of research themes were generated using the R package “bibliometrix” (version 4.4.1). This included analyses of annual publication volume, core source journals, productive countries and authors, and collaboration networks.

Network visualization and mapping of the intellectual structure were performed with VOSviewer (version 1.6.20) (25). Using the full counting method, the software was configured to map co-authorship networks (among countries, institutions, and authors) and keyword co-occurrence networks. Minimum threshold values for the number of documents or citations were adjusted for each analysis to generate clear and meaningful visualizations.

Temporal trend analysis and the detection of emerging research frontiers were conducted with CiteSpace (version 6.2. R4) (26, 27). Key parameters for the burst detection analysis of references and keywords were set as: time slicing (2000–2024, 1 year per slice), selection criteria (g-index, k = 25), and burst detection thresholds (*γ* = 1.0, minimum duration = 2 years). This allowed for the identification of pivotal publications and transiently high-interest topics within the field.

## 3 Results

### 3.1 Quantitative analysis of publications

**Figure 2:**
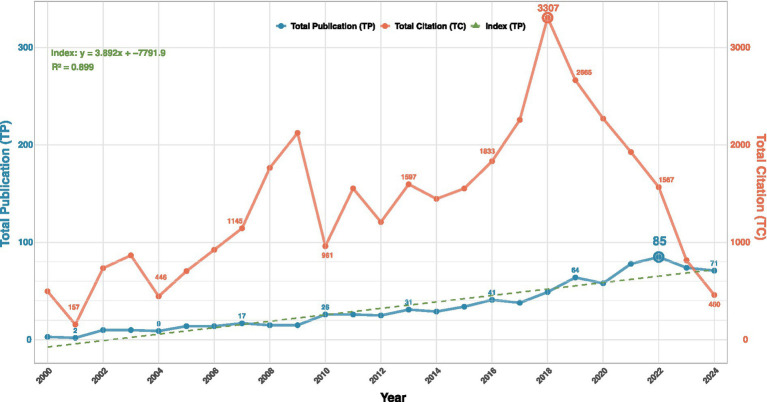
Annual total publications and total citations in terms of postoperative cognitive dysfunction associated with anesthesia from 2000 to 2024 (TP, Total publications; TC, Total citations).

A total of 923 publications on postoperative cognitive dysfunction associated with anesthesia were included in this analysis, comprising 697 “articles” (75.51%) and 226 “reviews” (24.49%). As depicted in Figure 2, the annual number of total publications showed a consistent upward trend from 2000 to 2022, reaching a peak of 85 publications in 2022. In contrast, total citations peaked earlier in 2018 at 3,307, followed by a rapid and sustained decline in subsequent years.

The publication trajectory can be divided into distinct phases. During the early period (2000–2009), annual publications remained below 20, with an average of only 36.92 per year across the entire study span. Between 2010 and 2020, publications exhibited fluctuating growth, followed by a slight decline after 2023. The total number of publications from 2018 to 2024 exceeded the cumulative output of the previous 17 years, indicating a recent surge in research activity. The selected 923 papers accumulated 34,791 citations, with an average of 37.69 citations per paper, reflecting good academic quality and suitability for in-depth analysis.

### 3.2 Analysis of countries and institutions

**Figure 3:**
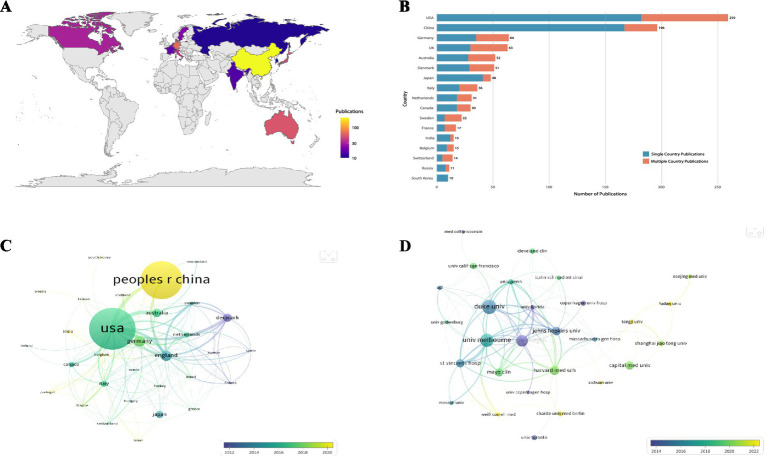
**(A)** Geographical distribution of countries/regions with more than 10 publications; **(B)** The proportion of national collaborations [SCP, Single country publications; MCP, Multiple country publications; MCP% = MCP/(SCP + MCP)]; **(C)** Overlay Visualization Map of countries/regions (minimum number of documents of country: 5); **(D)** Overlay Visualization Map of institutions (minimum number of documents of an institution: 5).

Publications originated from 59 countries/regions and 1,254 institutions based on VOSviewer analysis from 2000 to 2024. The United States ranked first with the highest number of publications (259, 28.06%), followed by China (196, 21.24%) and Germany (64, 6.93%) (Table 1, Figure 3A). The United States also led in total citations and total link strength. Specifically, Denmark, with only 51 publications, achieved the highest average citations per paper, indicating high research impact. The proportion of multiple-country publications reflects the degree of international collaboration. The United Kingdom showed the most frequent international cooperation in this field (Table 1, Figure 3B).

**Table 1:** The top 10 countries/regions in terms of postoperative cognitive dysfunction associated with anesthesia from 2000 to 2024.

Rank	Country	TP	TC	ACPP	TLS	SCP	MCP	MCP%	Institution	TP	TC	ACPP	TLS	PPY
1	USA	259	14,722	56.84	125	182	77	29.7	Duke University	39	3,423	87.77	67	2015
2	China	196	5,357	27.33	32	167	29	14.8	University Of Melbourne	36	3,291	91.42	87	2019
3	Germany	64	2,360	36.88	50	35	29	45.3	University Copenhagen Hospital	32	3,407	106.47	67	2011
4	UK	63	5,130	81.43	64	30	33	52.4	Harvard Medical University	25	2,363	94.52	64	2019
5	Australia	52	3,912	75.23	34	28	24	46.2	Johns Hopkins University	24	2,864	119.33	65	2021
6	Denmark	51	4,990	97.84	49	29	22	43.1	St Vincents Hospital	23	2,623	114.04	72	2011
7	Japan	48	1,112	23.17	8	41	7	14.6	Capital Medical University	23	433	18.83	4	2021
8	Italy	36	1,342	37.28	22	20	16	44.4	Mayo Clinical	22	1,420	64.55	49	2005
9	Netherlands	31	2,130	68.71	35	18	13	41.9	University California san Francisco	17	573	33.71	10	2019
10	Canada	30	1,652	55.07	17	18	12	40	University of Pennsylvania	15	1936	129.07	64	2011

TP, Total publications; TC, Total citations; ACPP, Average citations per paper; TLS, total line strength; SCP, Single country publications; MCP, Multiple country publications; MCP% = MCP/(SCP+MCP); PPY, Peak publication year.

Subsequently, we visualized the collaborative network of countries/regions with at least 10 publications (Figure 3C). The United States maintained extensive collaborations with Australia, China, and England. China also engaged actively with England and the United States. The most-cited article was published in the New England Journal of Medicine, suggesting that internationally collaborative studies tend to receive higher citations and appear in high-impact journals. Among them, China started publishing later than several other countries (Figure 3C).

The top 10 institutions by publication output were Duke University (39, 4.23%, USA), the University of Melbourne (36, 3.90%, Australia), and Copenhagen University Hospital (32, 3.47%, Denmark) (Table 1). Duke University also had the highest total citations. The University of Pennsylvania (USA) showed the highest ACPP, indicating strong academic influence, while St Vincent’s Hospital (Australia) had the highest total link strength. Charité – Universitätsmedizin Berlin and Copenhagen University Hospital were among the earliest institutions to experience a surge in publications, suggesting established research maturity in this area. Based on a threshold of at least 10 documents, 31 institutions were mapped in the collaboration network (Figure 3D). Copenhagen University Hospital emerged as one of the earliest and most collaborative institutions, followed by Duke University, the University of Melbourne, the University of Florida, and others.

### 3.3 Analysis of journals and co-cited journals

The 923 publications were published in 228 journals. The top 10 journals contributed 336 papers, accounting for 36.40% of the total. *British Journal of Anesthesia* published the most articles (47,5.09%), followed by *Anesthesia & Analgesia* (43, 4.66%) and *BMC Anesthesiology* (41, 4.44%) (Table 2). Remarkably, British Journal of Anesthesia also ranked first in CiteScore.

**Table 2:** The top 10 journals and co-cited journals in terms of postoperative cognitive dysfunction associated with anesthesia from 2000 to 2024.

Rank	Journal	TP	Hi	CS	TC	ACPP	TLS	IF	SPY	Co-cited Journal	TCC	TLS	IF
1	British Journal of Anaesthesia	47	159	14	4,668	14.7	690	9.2	2000	Anesthesiology	3,408	253,650	9.2
2	Anesthesia and Analgesia	43	214	11.4	3,148	13.3	574	9.2	2001	Anesthesia and Analgesia	2,326	181,353	9.2
3	BMC Anesthesiology	41	31	3.9	644	6	248	2.6	2001	British Journal of Anaesthesia	2060	141,890	9.2
4	Journal of Cardiothoracic and Vascular Anesthesia	37	76	4.6	933	6.5	240	5.1	2001	Annals of Thoracic Surgery	1,379	81,741	3.9
5	Anesthesiology	35	33	214	5,256	23.9	835	9.2	2002	Lancet	1,026	69,722	88.5
6	Current Opinion in Anesthesiology	28	27	61	622	5.2	145	2.1	2003	Acta Anaesthesiologica Scandinavica	1,007	59,138	2
7	Frontiers in Aging Neuroscience	27	25	55	352	7.9	213	3.5	2004	New England Journal of Medicine	872	66,727	78.5
8	Journal of Clinical Anesthesia	26	22	65	867	8.5	220	5.1	2004	Stroke	847	62,185	8.9
9	Annals of Thoracic Surgery	26	22	184	1,350	6.3	164	3.9	2005	Journal of the American Geriatrics Society	680	45,528	4.5
10	Minerva Anestesiologica	26	24	53	626	5.3	138	2.8	2009	Journal of Neurosurgical Anesthesiology	669	98,073	2.4

TP, Total publications; GI, G-index; ACPP, Average citations per; TLS, total line strength; IF, impact factor (from 2024 Journal Citation Reports); SPY, Start of publication year; TCC, Total Co-citations.

**Figure 4:**
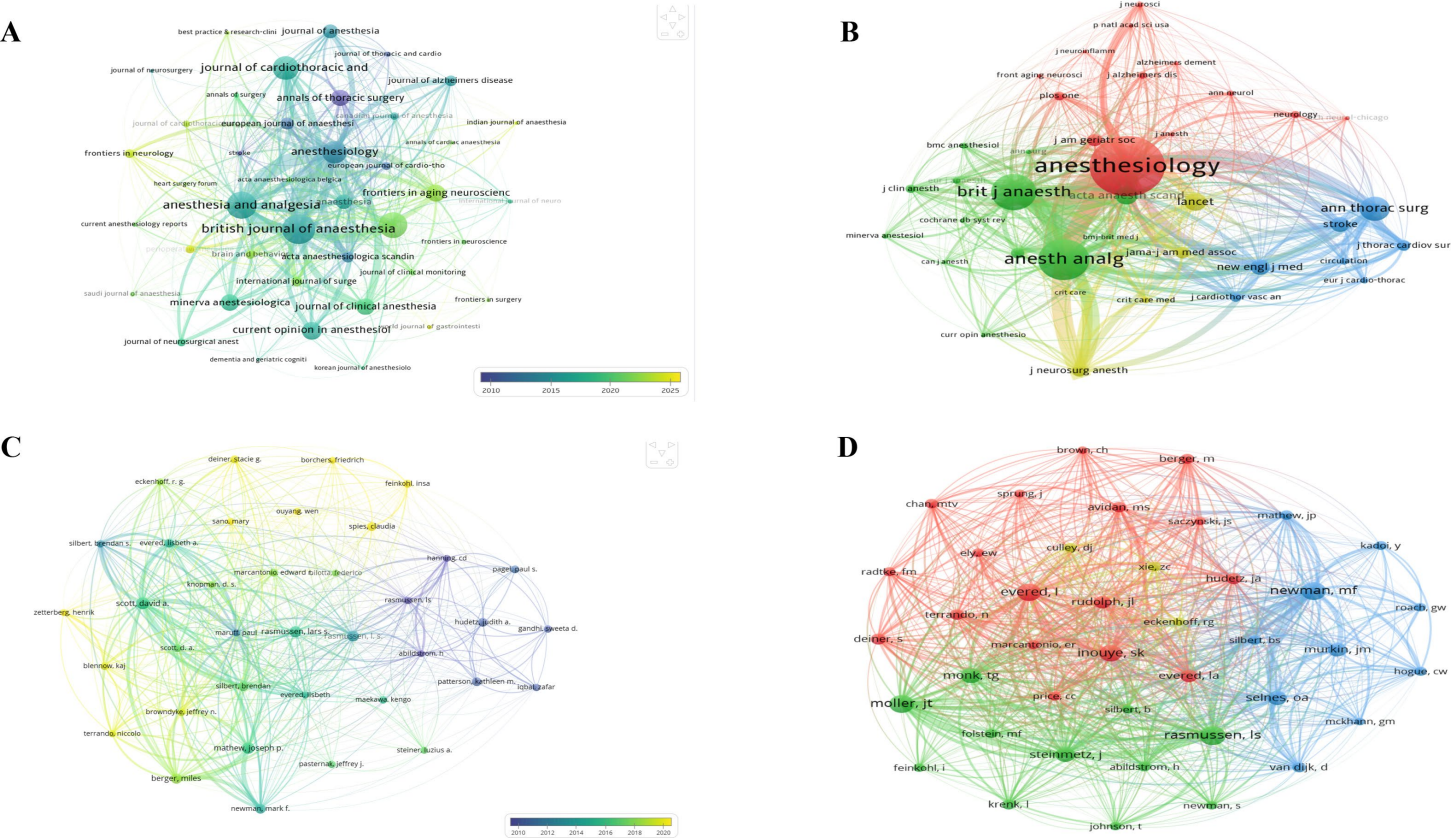
**(A)** Overlay visualization map of journals (minimum number off documents of a source: 5); **(B)** network visualization map of co-cited journal (minimum number of citations of a source: 200); **(C)** overlay visualization map of authors (minimum number of documents of an author: 6); **(D)** network visualization map of co-cited authors (minimum number of citations of an author: 60).

The journal with the highest academic influence was *Anesthesiology*, which exhibited the highest H-index (214) and Impact Factor (9.2) among the top 10, along with a strong average citation per paper and total link strength. We screened and mapped the journal network of 37 journals (Figure 4A), which revealed active collaborative relationships between *British Journal of Anesthesia* and *Anesthesia & Analgesia*.

As shown in Table 2, *Anesthesiology* was the most frequently co-cited journal, far exceeding other journals in co-citation frequency. Among co-cited journals, *Lancet* had the highest Impact Factor (88.5), followed by *New England Journal of Medicine* (78.5). Of the 4,761 co-cited journals, 38 had co-citation counts exceeding 200. As illustrated in Figure 4B, close co-citation relationships were observed among *Journal of Neurosurgical Anesthesiology*, *Annals of Thoracic Surgery*, and *PLOS ONE*.

### 3.4 Analysis of authors and co-cited authors

A total of 4,806 authors contributed to research in this field. David Anthony Scott (Australia) ranked first in total publications (18), total citations (1347), and total link strength (TLS = 644) (Table 3). Rasmussen, LS achieved the highest average citations per paper (ACPP = 86.7), indicating high per-paper impact, while Brendan Silbert exhibited a G-index of 17.75, reflecting sustained academic activity and influence in the field.

**Table 3:** The top 10 authors and co-cited authors in terms of postoperative cognitive dysfunction associated with anesthesia from 2000 to 2024.

Rank	Author	Country	TP	TC	TLS	ACPP	GI	SPY	Co-cited author	Country	TCC	TLS
1	David Anthony Scott	Australia	18	1,347	644	74.8	15.05	2004	Rasmussen, LS	Denmark	365	3,243
2	Joseph Patrick Mathew	USA	16	856	400	53.5	9.83	2002	Moller JT	Denmark	340	2,843
3	Rasmussen, LS	Denmark	15	1,301	267	86.7	17.20	2000	Newman, Mark F	USA	333	3,289
4	Miles Berger	USA	13	930	388	71.5	15.98	2013	Evered, Lisbeth	USA	323	3,134
5	Mark Francis Newman	USA	12	711	315	59.3	10.08	2002	Sharon K. Inouye	USA	290	2,956
6	Brendan Silbert	Australia	11	905	379	82.3	17.75	2008	Terri G Monk	USA	241	2,324
7	Evered, Lisbeth	USA	9	743	334	82.6	10.94	2004	Jacob Steinmetz	Denmark	222	2,302
8	Judith Ann Hudetz	USA	9	402	89	44.7	8.39	2007	Ola A. Selnes	USA	206	2,386
9	Spies, Claudia	Germany	9	216	20	24.0	6.07	2007	John M. Murkin	Canada	190	1,587
10	Stacie Grace Deiner	USA	8	494	133	61.8	10.18	2013	James L. Rudolph	USA	160	1939

TP, Total publications; TC, Total citations; ACPP, Average citations per paper; GI, G-index; SPY, Start of publication year; TCC, Total Co-citations; TLS, total line strength.

From the collaborative network (minimum number of documents of an author: 6) (Figure 4C), we observed active collaborations among different authors. Specifically, David Anthony Scott has worked closely with Brendan Silbert.

As shown in Table 3, eight authors were co-cited at least 200 times among the 54,451 co-cited authors. The most frequently co-cited author was Rasmussen, LS (365), followed by Moller, JT (340) and Newman, Mark *F* (333). The co-citation network map is presented in Figure 4D, which illustrates positive partnerships among multiple co-cited authors. As is shown by Figure 4D, Inouye, SK maintained active co-citation linkages with Rudolph, JL and Ely, EW.

From Table 3, we also note that Rasmussen, LS (Denmark) and Evered, Lisbeth (USA) appear among both the top 10 authors and the top 10 co-cited authors, underscoring their dual role as productive contributors and key intellectual references in this research domain.

### 3.5 Analysis of co-cited references

A total of 30,585 references were co-cited in research on postoperative cognitive dysfunction associated with anesthesia over the past 25 years. Table 4 presents the top 10 co-cited references based on co-citation frequency and relevant bibliometric information, each representing a milestone publication in this field. Among these ten references, six are clinical cohort or randomized controlled trials examining the incidence, predictors, and long-term outcomes of postoperative cognitive dysfunction; three are consensus statements or nomenclature recommendations aimed at standardizing research definitions and clinical assessment; and one is a systematic review synthesizing evidence on anesthetic techniques and cognitive outcomes.

**Table 4:** The top 10 co-cited references in terms of postoperative cognitive dysfunction associated with anesthesia from 2000 to 2024.

Rank	Title	Year	Country	Author	Journal	TCC	TLS	IF	Q	DOI
1	Longitudinal assessment of neurocognitive function after coronary-artery bypass surgery	2001	USA	Newman, Mark F	New England Journal of Medicine	194	374	78.5	1	10.1056/nejm200102083440601
2	Predictors of cognitive dysfunction after major noncardiac surgery	2008	USA	Monk, Terri G.	Anesthesiology	190	451	9.2	1	10.1097/01.anes.0000296071.19434.1e
3	Long-term Consequences of Postoperative Cognitive Dysfunction	2009	USA	Jacob Steinmetz	Anesthesiology	166	563	9.2	1	10.1097/aln.0b013e318195b569
4	The Assessment of Postoperative Cognitive Function	2001	Denmark	Lars Simon Rasmussen	Acta Anaesthesiologica Scandinavica	146	525	2	2	10.1034/j.1399-6576.2001.045003275.x
5	Recommendations for the nomenclature of cognitive change associated with anaesthesia and surgery-2018	2018	USA	Evered, Lisbeth	British Journal of Anaesthesia	119	282	9.2	1	10.1016/j.bja.2017.11.087
6	Does anaesthesia cause postoperative cognitive dysfunction? A randomised study of regional versus general anaesthesia in 438 elderly patients	2011	Denmark	Rasmussen, LS	Acta Anaesthesiologica Scandinavica	91	322	2	2	10.1034/j.1399-6576.2003.00057.x
7	BIS-guided Anesthesia Decreases Postoperative Delirium and Cognitive Decline	2002	Hong Kong	Matthew T. V. Chan	Journal of Neurosurgical Anesthesiology	86	244	2.4	2	10.1097/ana.0b013e3182712fba
8	Postoperative Cognitive Dysfunction Is Independent of Type of Surgery and Anesthetic	2011	Australia	Evered, Lisbeth	Anesthesia and Analgesia	81	317	9.2	1	10.1213/ane.0b013e318215217e
9	Postoperative cognitive dysfunction in middle-aged patients	2002	USA	Johnson, Trevor	Anesthesiology	76	303	9.2	1	10.1097/00000542-200206000-00014
10	Type and severity of cognitive decline in older adults after noncardiac surgery	2008	USA	Catherine C. Price	Anesthesiology	67	255	9.2	1	10.1097/01.anes.0000296072.02527.18

TC, Total Co-citations; IF, impact factor (from 2024 Journal Citation Reports); Q: from 2024 Journal Citation Reports.

As is shown in Table 4. Five of the ten publications resulted from international collaborations, including partnerships between the United States and Denmark, the United States and Australia, and multinational consortia. All of these collaboratively authored papers were published in high-impact journals, reflecting the global and interdisciplinary nature of research in this area.

The article by Newman et al. (28) received the highest co-citation frequency and is widely regarded as a foundational study that established longitudinal neurocognitive assessment following cardiac surgery. The paper with the highest impact factor (78.5) was published in the New England Journal of Medicine, providing seminal evidence on cognitive outcomes after coronary-artery bypass grafting.

**Figure 5:**
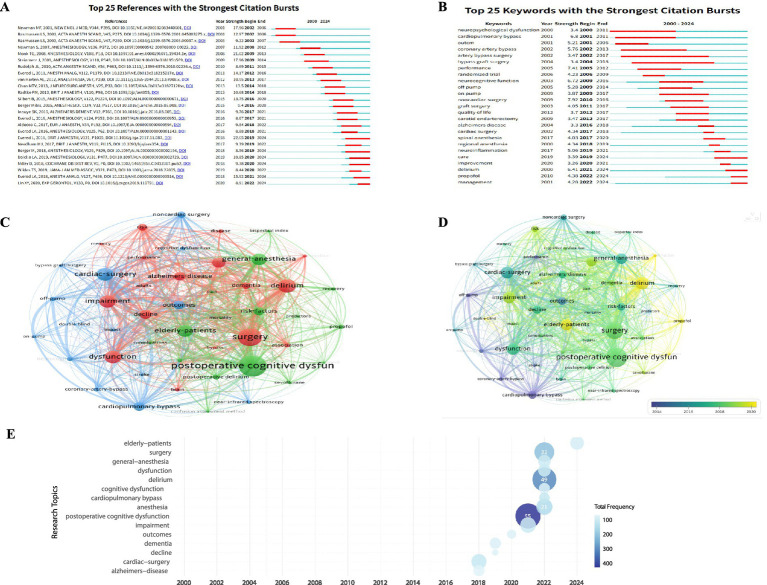
**(A)** Top 25 references with the strongest citation bursts; **(B)** top 25 keywords with the strongest citation bursts; **(C)** network visualization map of keywords occurrence analysis (minimum number of occurrences of a keyword: 25); **(D)** overlay visualization map of keywords occurrence analysis (minimum number of occurrence of a keyword: 25). **(E)** Trend topic frequency map showing the evolution of research hotspots over time via External validation using PubMed database.

As shown in Figure 5A, the citation burst strength of the top 25 references ranged from 9.22 to 22.65, with bursts occurring between 2002 and 2024. The reference with the strongest citation burst was the 2018 study by Evered et al. (21), which experienced a prominent burst from 2019 to 2024, highlighting its giant and lasting influence in shaping research directions.

### 3.6 Analysis of keywords

Through citation burst, co-occurrence clustering, and trend topic analysis, we identified key research themes in postoperative cognitive dysfunction associated with anesthesia. Figure 5B shows the top 25 keywords with the strongest citation bursts from 2000 to 2024. Early bursts, including “Noncardiac Surgery” (strength 7.92, 2010–2016), “Performance” (strength 7.41, 2005–2012), and “cardiopulmonary bypass” (strength 6.8, 2001–2011), reflect an initial focus on surgical types and neurocognitive assessment. Recent burst keywords especially “neuroinflammation,” “delirium,” “propofol,” and “management” indicate a shift toward mechanisms, pharmacology, and clinical management.

Keyword co-occurrence clustering (threshold: 50 occurrences) revealed three thematic clusters (Figures 5C,D). The red cluster includes clinical and surgical context terms like “surgery,” “cardiac surgery,” “noncardiac surgery,” “elderly patients,” and “outcomes,” highlighting perioperative epidemiology and patient-oriented research. The blue cluster centers on pathophysiology and mechanisms with terms such as “neuroinflammation,” “Alzheimer’s disease,” “dementia,” and “risk factors,” indicating growing interest in neurodegenerative links to postoperative cognitive decline. The green cluster focuses on anesthetic techniques and interventions including “general anesthesia,” “regional anesthesia,” “propofol,” and “management,” reflecting research on anesthesia modulation and perioperative care.

The overlay visualization (Figure 5D) illustrates temporal evolution, with color intensity corresponding to average activity year. Early-stage keywords like “off-pump,” “neurocognitive function,” “coronary-artery bypass,” and “cardiopulmonary bypass” emphasize surgical techniques and cognitive assessment. Mid-stage terms including “noncardiac surgery,” “general-anesthesia,” “cognitive dysfunction,” “Alzheimer’s disease,” and “postoperative delirium” reflect broader research into surgical diversity, anesthesia modalities, and neurodegenerative associations. Recent keywords including “delirium” and “propofol” highlight current focus on acute syndromes and pharmacologic interventions.

Trend topic analysis using the R package “bibliometrix” confirmed these patterns. High peak-frequency keywords map (Figure 5E) in recent years—“postoperative cognitive dysfunction,” “delirium,” and “surgery”—underscore the continued centrality of core clinical phenotypes and procedural contexts, supported by terms like “anesthesia” and “impairment,” reflecting sustained research into perioperative entities and functional outcomes.

### 3.7 Validation using external databases

To ensure the robustness and cross-platform generalizability of the analytical results derived from the Web of Science Core Collection, we conducted external validation using the PubMed database. A parallel search strategy and screening process identical to that applied in the WoSCC analysis was implemented in PubMed, yielding 318 relevant publications for systematic comparative analysis.

**Figure 6:**
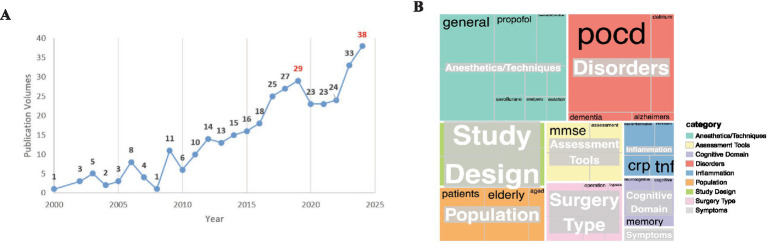
**(A)** Annual publications using PubMed datasets from 2000 to 2024; **(B)** Keywords frequency maps using PubMed datasets.

The initial comparative analysis focused on the temporal evolution of the field. A direct year-by-year comparison of publication volumes (Figure 6A) revealed a congruent growth trajectory between the two databases. Both platforms exhibited low output in the early 2000s, followed by a sustained and parallel increase beginning around 2010, with publication peaks occurring in the 2022–2023 period. This synchronized temporal pattern provides strong preliminary evidence that the observed expansion of research activity is a robust, database-independent trend.

Quantitative assessment of thematic consistency between the two databases yielded a Jaccard similarity coefficient of 0.68 for the overlap of the top 50 keywords, indicating substantial agreement in core research themes. The overlapping keywords included ‘postoperative cognitive dysfunction,’ ‘delirium,’ ‘neuroinflammation,’ ‘cardiac surgery,’ ‘elderly patients,’ ‘general anesthesia,’ and ‘risk factors.

The results demonstrate a high degree of consistency between the two databases in the structural organization of core research themes (Figure 6B, Table 5) Studies in both platforms consistently revolve around several interrelated academic dimensions: at the clinical level, research focuses on comparing anesthetic techniques, assessing the impact of different surgical procedures, and optimizing integrated perioperative management strategies; at the pathophysiological level, the central role of neuroinflammation is widely acknowledged, with investigations extending into the delirium–postoperative cognitive dysfunction continuum and its potential links with neurodegenerative diseases; and at the population level, elderly surgical patients are consistently identified as a high-risk group, with cognitive outcomes and optimal timing of intervention representing shared research priorities. This structural alignment confirms the validity of the bibliometric mapping based on WoSCC. Furthermore, PubMed data highlight supplementary methodologically-oriented themes, particularly in the refinement of study designs—such as rigorous implementation of randomized controlled trials, advances in systematic review and meta-analysis methodologies, and standardized application of neuropsychological assessment tools—as well as in the more pronounced emphasis on the relationship between perioperative pain management and cognitive outcomes. These themes collectively enrich the research landscape, reflecting an evolving trajectory from descriptive clinical observation toward mechanistic exploration and translational practice. The consistency of findings across databases reinforces the stability of the identified research hotspots and trends, thereby enhancing the credibility and scholarly value of the conclusions drawn in this study.

**Table 5:** Comparative analysis of high-frequency keywords between web of science and PubMed corpora.

Aspect	WoS keywords	PubMed keywords
Anesthetic techniques	General anesthesia, regional anesthesia, propofol	Anaesthesia, general, propofol, sedation, spinal
Surgical types	Cardiac surgery, noncardiac surgery, cardiopulmonary bypass	Cardiac, CPB, operation
Diseases/disorders	Delirium, Alzheimer’s disease, dementia	Delirium, dementia, POCD
Pathophysiological mechanisms	Neuroinflammation, risk factors	Neuroinflammation, inflammatory
Population characteristics	Elderly patients	Elderly, patients
assessment methods	Performance	Assessment, score

## 4 Discussion

Through an analysis of 923 publications spanning the years 2000 to 2024, this bibliometric study synthesizes and visualizes data concerning anesthesia-associated postoperative cognitive dysfunction (POCD). It aims to offer a comprehensive overview of key contributors, delineate the present research landscape, and identify emerging topics and future trajectories within the field.

### 4.1 General information

This bibliometric analysis reveals steady growth in anesthesia-associated POCD research over the past quarter-century, with publication activity accelerating significantly after 2018. This surge reflects the field’s evolution from initial clinical observations to a major focus within perioperative medicine, driven by an aging surgical population and growing awareness of POCD’s long-term implications. To substantiate the robustness of our findings from the Web of Science Core Collection (WoSCC), we conducted a focused validation using the PubMed database. Comparative analysis of publication volumes between 2000 and 2024 revealed congruent growth trajectories, with research peaking around 2022–2023. Independent keyword co-occurrence analysis on PubMed reproduced the three primary thematic clusters identified in WoSCC. This convergence across two independently indexed databases confirms that our findings reflect the field’s genuine evolution rather than database-specific artifacts.

Geographically, the United States dominates in productivity, influence, and collaborative network strength, with institutions like Duke University and the University of Florida serving as central hubs. China has emerged as a major contributor in publication volume, though its per-paper citation impact is still developing. Nations with longer research histories, such as Denmark and Germany, demonstrate exceptionally high scholarly impact per publication. Regarding individual scholarly impact, key figures have shaped the field. Lars S. Rasmussen has defined POCD’s epidemiology through rigorous clinical studies. Lisbeth Evered has led efforts to standardize nomenclature and promote biomarker research. Terri G. Monk has examined anesthetic management and patient-specific risk factors. Their work forms the essential conceptual framework of contemporary POCD research.

The field’s intellectual foundation is crystallized in its most co-cited references. The longitudinal study by Newman et al. (28) in The New England Journal of Medicine first established the significance of cognitive decline after cardiac surgery. The consensus statement by Evered et al. (21) proposed a unified nomenclature, addressing longstanding definitional challenges and guiding the field’s future direction.

### 4.2 Evolving research hotspots and thematic trends

**Figure 7:**
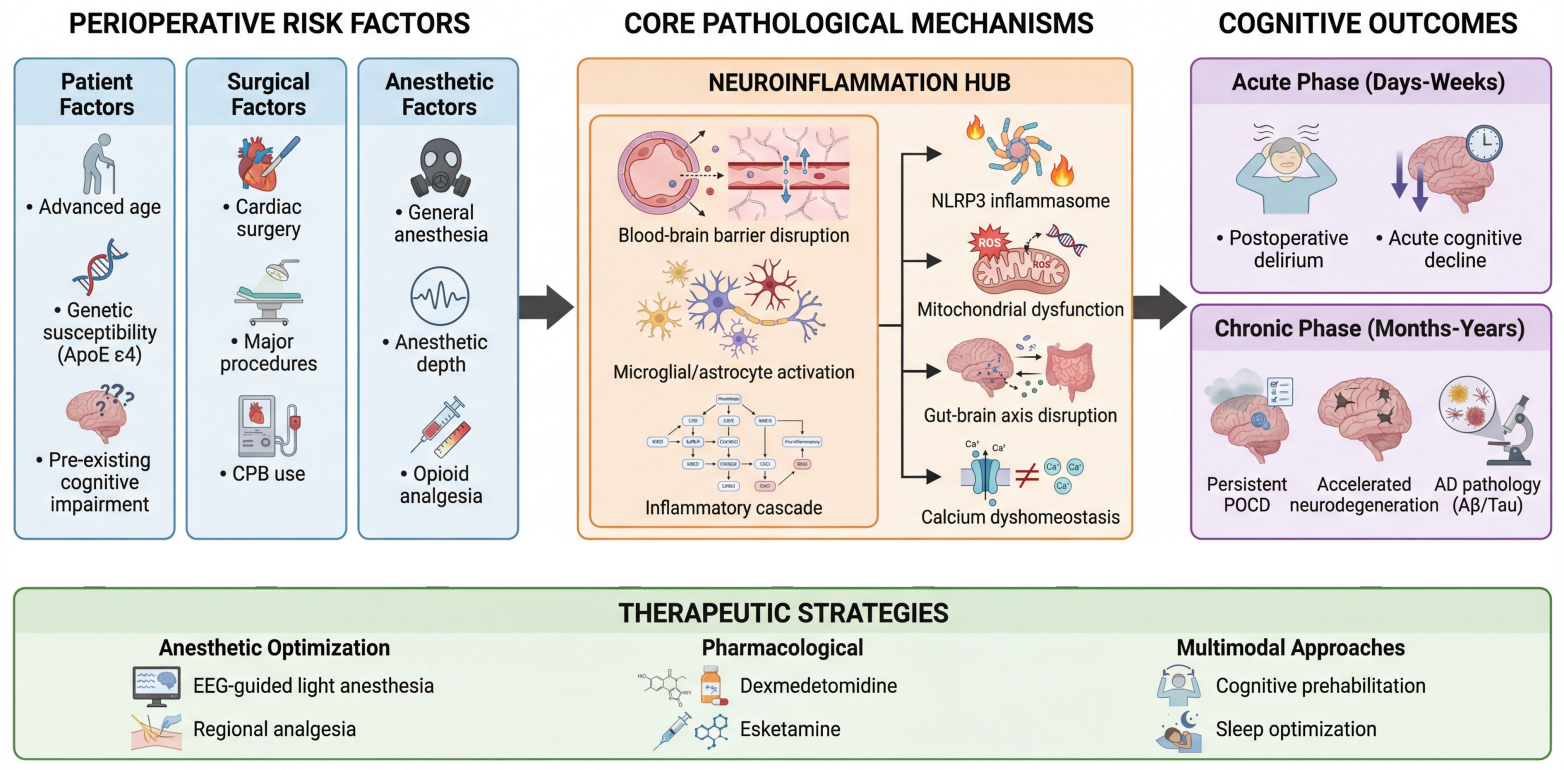
Three-tier framework of POCD pathogenesis and intervention.

Analysis of keyword evolution reveals a clear maturation trajectory in POCD research, progressing from descriptive epidemiology to mechanism-focused inquiry and finally to translational exploration. This development represents a convergent pathway where clinical observations generate mechanistic hypotheses, which in turn inform potential therapeutic strategies. At the core of this evolution, neuroinflammation has emerged as the unifying central theme bridging clinical observations and preclinical mechanisms. The Three-tier framework is shown in Figure 7.

#### 4.2.1 Clinical-translational research

Clinical research on POCD has undergone significant evolution, shifting from initial questions of “who is at risk” and “what happens” to deeper inquiries of “how to predict and ultimately prevent it.”

This transformation first manifests in the refined characterization of risk. Early studies established the foundation for non-modifiable patient risk factors such as advanced age, lower educational attainment, and pre-existing cognitive impairment (7, 10), while identifying specific high-risk surgical contexts including cardiac procedures (2, 28). Subsequent research has substantiated the profound long-term consequences of POCD—encompassing increased mortality, functional decline, and diminished quality of life—thereby elevating its status from a postoperative complication to a significant public health concern (4, 5). Current research frontiers are moving beyond static risk assessment toward dynamic prediction, focusing on developing validated clinical risk scores and identifying reliable biomarkers to enable preemptive, personalized management (29, 30).

Within this context, a crucial conceptual breakthrough has been the formulation of the “perioperative neurocognitive continuum.” The research community no longer views postoperative delirium and POCD as isolated events but recognizes them as interconnected points along the same spectrum (3). The surge in research attention to “delirium” reflects intensified investigation into its role as both a robust predictor and potential prodrome of longer-term cognitive decline. This continuum is biologically plausible, as both acute delirium and chronic dysfunction are believed to share common pathological substrates, thereby bridging acute care and long-term cognitive outcomes (6).

Within this framework, the modulation of modifiable perioperative factors naturally emerges as a persistent research focus. The evidentiary landscape surrounding anesthetic and analgesic management is complex and evolving. While early systematic reviews and large-scale RCTs found no definitive difference in POCD incidence between regional and general anesthesia (11, 31), recent meta-analyses focusing on specific high-risk contexts (e.g., hip fracture surgery) suggest potential advantages for regional techniques (22, 32). Similarly, comparative studies of intravenous versus inhalational anesthetics remain active, with recent meta-analyses and randomized controlled trials demonstrating context-dependent outcomes, indicating that the optimal choice may vary according to patient population and surgical type (33–38). In contrast, evidence supporting the modulation of anesthetic depth is more robust: multiple randomized trials have confirmed that lighter anesthesia guided by electroencephalographic monitoring can reduce the risk of POCD (9, 39, 40).

Furthermore, postoperative analgesia itself has been repositioned as a direct cognitive protective strategy. Research indicates that effective multimodal analgesia, particularly regimens that minimize systemic opioid exposure through techniques such as regional nerve blocks, can mitigate surgical stress and inflammatory responses, thereby directly reducing POCD incidence and providing critical clinical support for the neuroinflammatory hypothesis (12, 41–43).

Reflecting a holistic understanding of the multifactorial etiology of POCD, the research frontier is expanding from single pharmacological or technical interventions to exploring comprehensive multimodal prevention strategies. These encompass cognitive prehabilitation, sleep optimization, and complementary therapies such as transcutaneous electrical acupoint stimulation, with the overarching goal of comprehensively enhancing patients’ physiological and cognitive resilience during the perioperative period (23, 44). This shift from seeking a single neuroprotective agent to implementing integrated perioperative brain health strategies represents the current forefront of clinical-translational research.

#### 4.2.2 Preclinical-mechanistic research

While clinical research delineates the problem and seeks solutions, preclinical investigations concurrently dissect its biological essence. Within this domain, neuroinflammation has been established as the unequivocal central pathological hub (13, 14). The “double-hit” model—where surgical trauma combined with anesthetic exposure disrupts the blood–brain barrier and activates glial cells—provides a robust conceptual framework.

Current research is conducting fine-grained dissection of this neuroinflammatory cascade at multiple levels. Key mechanisms at the forefront of investigation include the activation of the NLRP3 inflammasome and associated pyroptosis, with recent studies further indicating that upstream mitophagy dysfunction serves as a critical trigger (45). Novel signaling molecules such as Sarm1 have been confirmed as indispensable mediators in anesthesia-induced neuroinflammation (46). Concurrently, dysregulation of intracellular calcium homeostasis in aged neurons—a fundamental vulnerability exacerbated by anesthetic exposure—has garnered significant attention (47). Furthermore, the role of the gut-brain axis and its microbiota in modulating peripheral and central immune responses represents a rapidly expanding research dimension, adding a crucial systemic perspective to the pathophysiology (48).

A vital line of mechanistic inquiry is dedicated to bridging POCD with neurodegenerative disorders, especially Alzheimer’s disease (AD). Research efforts are elucidating how inflammation may accelerate amyloid-beta and tau pathology, and how anesthetic agents might directly influence AD-related molecular pathways (16, 17, 49). This work provides a biological foundation for observed clinical associations and elevates the significance of POCD from a transient event to a potential modifier of long-term cognitive trajectory.

The elucidation of these mechanisms directly guides the discovery of novel therapeutic targets. Preclinical models are utilized not only to validate the neuroprotective mechanisms of established agents like dexmedetomidine but also to explore new candidates like.

Esketamine, melatonin, and magnesium sulfate (24, 50–52). The ultimate objective is to translate these mechanistic insights into targeted therapies amenable to clinical testing.

Collectively, the research foci within the POCD domain are dynamic and intricately interconnected. Clinical research continuously refines the problem definition and evaluates real-world solutions, while preclinical investigation persistently unravels the underlying biological principles and identifies novel intervention targets. The convergence of these two axes, united by a shared emphasis on neuroinflammation, the delirium-cognition continuum, and multimodal interventions—represents the pivotal frontier where the most promising breakthroughs for preventing and treating this significant postoperative syndrome are anticipated to emerge.

#### 4.2.3 From neuroinflammation to neuroprotection: translational progress and bottlenecks

The elucidation of neuroinflammatory mechanisms has directly guided the discovery and clinical evaluation of neuroprotective strategies. Dexmedetomidine, an α2-adrenoceptor agonist, reduces pro-inflammatory cytokines and activates the cholinergic anti-inflammatory pathway; multiple meta-analyses support its efficacy in reducing postoperative delirium and POCD, particularly when applied postoperatively in cardiac surgery patients (53–55). Ketamine and its S-enantiomer esketamine exhibit NMDA receptor antagonism with anti-inflammatory effects, though clinical evidence remains mixed. A recent meta-analysis reported that esketamine significantly reduces the risk of perioperative neurocognitive disorders (RR: 0.53), yet the certainty of evidence was low, and a separate RCT found no reduction in POCD incidence (56, 57). Propofol possesses antioxidant properties and suppresses microglial activation, but meta-analyses comparing propofol to sevoflurane have demonstrated no definitive superiority in overall POCD incidence (55). Regional anesthesia techniques attenuate surgical stress responses and have shown benefit in specific contexts such as hip fracture surgery (58).

### 4.3 Limitations

This study has several limitations. First, focusing exclusively on English-language articles in the Web of Science Core Collection may have omitted relevant studies in other languages or databases. Second, although we targeted ‘anesthesia-associated’ POCD, the included studies inevitably confound the effects of anesthesia with those of surgical trauma, as the ‘double-hit’ model suggests that surgical inflammation may be the predominant driver. Third, methodological heterogeneity in POCD diagnostic criteria may have influenced keyword clustering and citation patterns. Lastly, time lags in citation counts may underestimate the influence of recent high-quality studies. Despite these limitations, our inclusion of the most recent and widely cited literature provides a robust and timely overview of the POCD research landscape.

## 5 Conclusion

This bibliometric analysis delineates the evolution and current trajectory of research on anesthesia-associated postoperative cognitive dysfunction (POCD). The field has progressed from early epidemiological studies to a mechanistic and translational focus, with neuroinflammation established as a central pathological driver. Clinically, efforts are directed toward risk stratification, understanding the delirium-cognition continuum, and developing multimodal prevention strategies. Methodologically, the integration of advanced neuroimaging, omics, and neural circuit techniques is refining mechanistic insights and biomarker discovery. Despite advances, key challenges persist, including elucidating precise mechanisms, validating clinical prediction models, and establishing effective interventions through rigorous trials. Future research will likely further integrate clinical and multi-omics approaches to bridge perioperative stress, neuroinflammation, and cognitive outcomes, guiding targeted neuroprotective strategies.

## Data Availability

The original contributions presented in the study are included in the article/supplementary material, further inquiries can be directed to the corresponding author.
